# Viral-mediated activation and inhibition of programmed cell death

**DOI:** 10.1371/journal.ppat.1010718

**Published:** 2022-08-11

**Authors:** Shayla Grace Verburg, Rebecca Margaret Lelievre, Michael James Westerveld, Jordon Marcus Inkol, Yi Lin Sun, Samuel Tekeste Workenhe

**Affiliations:** Department of Pathobiology, Ontario Veterinary College, University of Guelph, Guelph, Canada; NYU Langone Health, UNITED STATES

## Abstract

Viruses are ubiquitous intracellular genetic parasites that heavily rely on the infected cell to complete their replication life cycle. This dependency on the host machinery forces viruses to modulate a variety of cellular processes including cell survival and cell death. Viruses are known to activate and block almost all types of programmed cell death (PCD) known so far. Modulating PCD in infected hosts has a variety of direct and indirect effects on viral pathogenesis and antiviral immunity. The mechanisms leading to apoptosis following virus infection is widely studied, but several modalities of PCD, including necroptosis, pyroptosis, ferroptosis, and paraptosis, are relatively understudied. In this review, we cover the mechanisms by which viruses activate and inhibit PCDs and suggest perspectives on how these affect viral pathogenesis and immunity.

## Introduction

Programmed cell death (PCD) plays a fundamental role in organismal development, infectious disease pathogenesis, immune regulation, and tissue homeostasis. Dysregulated PCD is associated with a wide variety of diseases, including immunological and developmental disorders, neurodegeneration, and cancer. Hence, understanding the mechanisms of PCD regulation is essential to devise strategies to manipulate PCD to manage several disorders.

PCD is initiated after cells sense genotoxic, metabolic, and infectious insults. During virus infection, sensing of viral-derived nucleic acids can initiate signaling to activate PCD. To a varying degree, viruses utilize the infected cell transcription and translation machinery to complete their life cycle; thus, produce nucleic acid replication intermediates that are identified as non-self by a variety of host-encoded nucleic acid sensors [1]. Detection of nucleic acids activates a plethora of interconnected antiviral pathways including activation of the interferon (IFN)-mediated antiviral state, translational arrest, and PCD. In a concerted approach, these processes restrict virus propagation until the development of virus-specific adaptive immunity [1]. Recently, it has been shown that emission of immunomodulatory secretomes after PCD shapes the development of adaptive immunity. There are several elegant reviews that document the immunogenicity of PCD [2].

While the mechanisms by which pathogen-associated molecular pattern (PAMP) sensing activates antiviral state have been extensively studied, so much remains unknown about how viral sensing regulates new modalities of PCD. Virus-infected cells activate nucleic acid–mediated antiviral responses along with a variety of endoplasmic reticulum (ER) [3], mitochondrial [4] and oxidative stress [5], and translational arrest that can directly or indirectly influence the induction of PCD [6]. In addition, several virus families encode proteins to inhibit the function of nucleic acid sensors [7], various antiviral factors such as IFN-stimulated genes (ISGs), and even proteins involved in the initiation and execution of PCD [8]. Hence, it is still unknown what types of nucleic acid receptors are directly involved in the induction of newly emerging PCD types. In this review, we provide literature on the mechanisms by which viruses activate and inhibit PCD and how that affects viral pathogenesis and host immunity.

### Sensing of viral-associated nucleic acids

Availability, localization, and structure of viral-associated nucleic acids in infected cells provide host-encoded sensors to selectively identify non-self-nucleic acids. Viral-associated nucleic acids are sensed by cell surface–expressed (Toll-like receptor 3 (TLR3)), endosome-localized (TLR3, TLR7, TLR8, TLR9), and cytoplasmic sensors (retinoic acid–inducible gene I (RIG-I)/melanoma differentiation–associated gene 5 (MDA5)/nucleotide-binding oligomerization domain-like receptors (NLRPs) such as NLRP1 [9], NLRP6-DHX15 [10], NLRP9-DHX9 [11]/cyclic GMP–AMP synthetase (cGAS) stimulator of IFN genes (STING), absent in melanoma 2 (AIM2). Viral RNA is mainly recognized by TLR3 and RIG-I-like helicases (RLHs) (RIG-I/MDA5), whereas DNA is sensed by cGAS, AIM2-like receptors (ALRs), and TLR9. While most RNA sensors are well established, numerous DNA receptors continue to be discovered, including the human DNA sensor DNA-PK that functions independent of STING [12]. Species and cell-specific effects may account for the plethora of DNA sensors postulated, for instance, deletion of the ALR locus did not affect the IFN response to cytosolic DNA, dispelling the role of ALR in mice [13].

Most nucleic acid receptors, including the TLR family, the RLHs, and cGAS-STING, directly or indirectly induce transcription factors, including IFN-regulatory factor 3 (IRF3) and nuclear factor-κB (NF-κB), which up-regulate the expression of antiviral effector proteins, chemokines, and cytokines. RLHs and STING-cGAS are major nucleic acid sensors that activate phosphorylation-induced oligomerization of adaptor proteins mitochondrial antiviral-signaling (MAVS) and STING, respectively. Oligomerized MAVS and STING are scaffolding proteins closely associated with the IkB kinase (IKK)-related kinases TANK-binding kinase (TBK1) and IKK-e, which are responsible for phosphorylation of IRF3/7 [14–16], and IKK-b and IKK-a, which phosphorylate NF-kB [17]. IRF3/7 and NF-kB are transcription factors that bind regions in the promoter to up-regulate the expression of IFNs and proinflammatory cytokines, respectively [18,19].

Binding of secreted IFN-a/b to IFN receptors activates the associated Janus kinases (JAKs) tyrosine kinase 2 (TYK2) and JAK1, which phosphorylate both signal transducer and activator of transcription 1 (STAT1) and STAT2. STAT1, STAT2, and IRF9 form the IFN-stimulated gene factor 3 (ISGF3) complex that translocates to the nucleus to bind IFN-stimulated response elements (ISREs) and promote the expression of hundreds of ISGs. ISGs have a variety of functions including antiviral defense and initiation of adaptive immunity and collectively create a nonpermissive antiviral state [20].

Although not a focus of this review, there are also nucleic acid receptors with direct antiviral activity, including double-stranded RNA (dsRNA)-activated protein kinase R, 2′5′oligoadenylate synthetase 1 (OAS1) and adenosine deaminase acting on RNA 1 (ADAR1). OAS1 and ADAR1 recognize foreign nucleic acids to directly act on viral RNA by either inhibiting translation or degradation of the target RNA.

### 1. Mechanisms of programmed cell death activation after virus infection

In addition to the secretion of proteins with direct antiviral effect, nucleic acid sensing pathways involving RLHs, TLRs, Z-DNA/RNA binding protein 1 (ZBP1)/DNA-dependent activator of IFN-regulatory factors (DAI), NLRPs, and cGAS-STING play a role in the activation of apoptosis, necroptosis, and ferroptosis. Overall, the different types of PCD activated during virus infection involve distinct signaling pathways, some exclusively relying on caspases (apoptosis and pyroptosis) and others depending on kinases (necroptosis) and lipid peroxidation sensors (ferroptosis). In the following sections, we present a concise summary of how viruses activate the various PCD pathways.

### Mechanisms of apoptosis induction during virus infection

**Fig 1:**
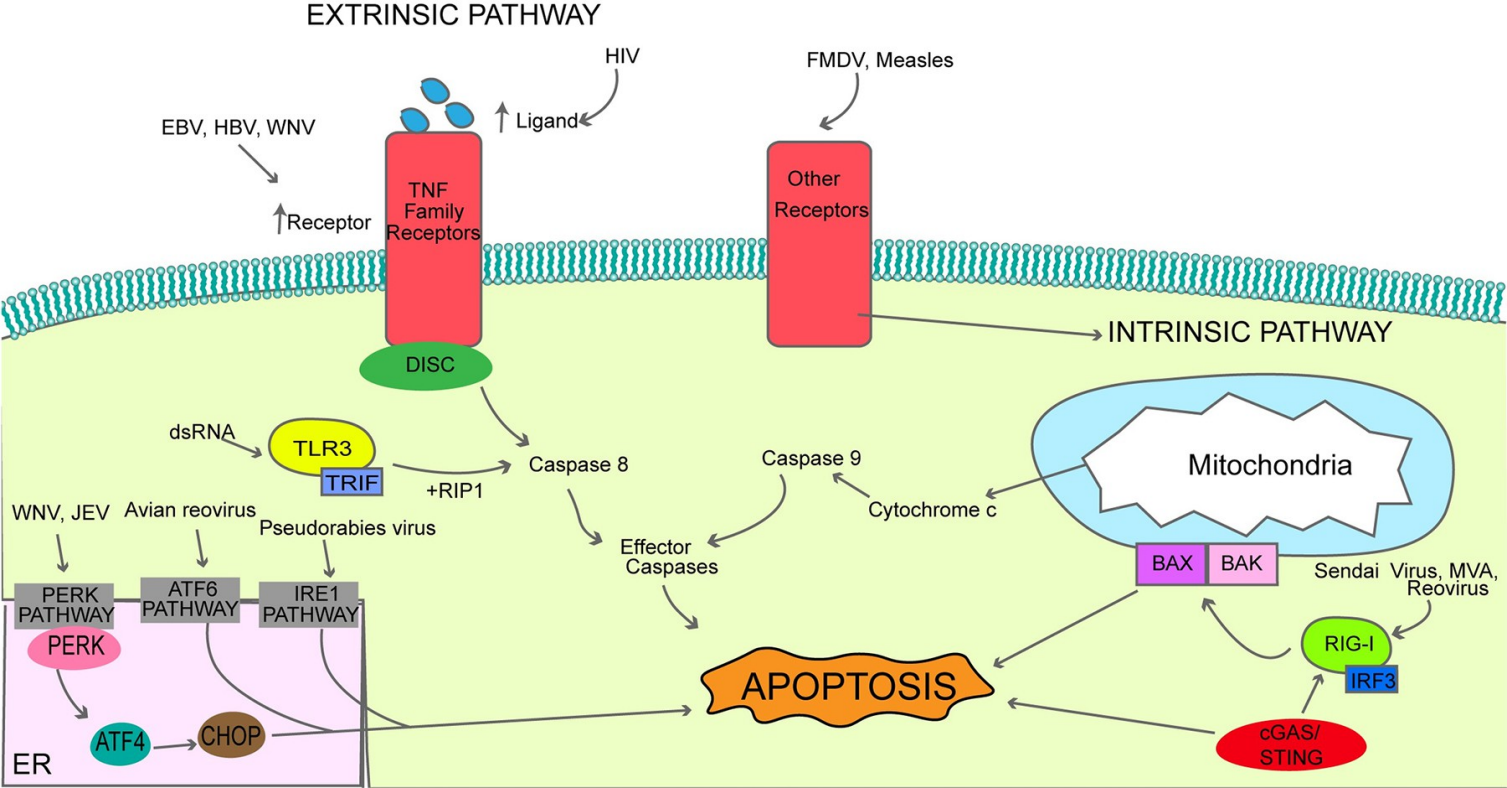
A summary of the major components of the extrinsic and intrinsic pathways of apoptosis induction following viral infection. In response to viral infection, the extrinsic pathway is initiated by death receptors following stimulation by the TNF proteins, resulting in downstream signaling and caspase-8 activation. The intrinsic pathway of apoptosis arises from proapoptotic stimuli, subsequently initiating mitochondrial membrane permeabilization and activation of caspase-9. Both pathways converge at the terminal activation of caspase-3 that executes apoptosis. ATF4, activating transcription factor 4; ATF6, activating transcription factor 6; BAK, BCL-2 antagonist or killer; BAX, BCL-2-associated X protein; cGAS/STING, cyclic GMP–AMP synthetase/stimulator of IFN genes; CHOP, CCAAT-enhancer-binding protein homologous protein; DISC, death-inducing signaling complex; EBV, Epstein–Barr virus; ER, endoplasmic reticulum; FMDV, Foot-and-mouth disease virus; HBV, hepatitis B virus; IRE1, inositol-requiring enzyme 1; IRF3, IFN-regulatory factor 3; JEV, Japanese encephalitis virus; MVA, modified vaccinia virus; PERK, PKR-like ER kinase; RIG-I, retinoic acid-inducible gene I; TLR3, Toll-like receptor 3; TNF, tumor necrosis factor; TRIF, Toll/IL-1 receptor domain-containing adapter inducing IFN-beta; WNV, West Nile virus.

Apoptosis is one of the most researched viral-induced PCD. In virus-infected cells, apoptosis can be initiated via the extrinsic or intrinsic pathway (Fig 1; [21]). In the extrinsic pathway, apoptosis begins after activation of the death domain receptors by the tumor necrosis factor (TNF) family of proteins including Fas activated by the FasL ligand, TNF-related apoptosis-inducing ligand (TRAIL) receptors 1 and 2 activated by TRAIL, and TNF receptor 1 (TNF-R1) activated by TNF [21,22]. Ligand binding to Fas and TRAIL receptors leads to oligomerization of the death receptors and assembly of the death-inducing signaling complex (DISC) consisting of death receptor, the adaptor molecule FADD (FAS-associated with a death domain), procaspase-8, procaspase-10, and cellular FADD-like interleukin-1β (IL-1β)-converting enzyme (FLICE)-like inhibitory protein (c-FLIP). c-FLIP has 2 isoforms, long and short, which control the activation of caspase cascade that emanates from caspase-8-mediated apoptotic and nonapoptotic signaling. In contrast to Fas and TRAIL death receptors, TNF-R1-mediated apoptotic signaling is complex and recruits TNF-R-associated protein with death domain (TRADD) as an adaptor protein, which subsequently recruits FADD, TNF-associated factor-2 (TRAF-2), receptor-interacting protein (RIP), and RIP-associated IL-1β-converting enzyme homolog (ICH-1)/cell death protein-3 (CED-3)-homologous protein with a death domain (RAIDD). FADD then binds and activates the extrinsic apoptosis initiator caspase-8, which subsequently activates the effector caspases-3/6/7 [23].

In the intrinsic pathway, several proapoptotic stimuli result in mitochondrial outer membrane permeabilization [24,25]. Mitochondrial permeabilization permits the release of mitochondrial intermembrane space proteins, such as cytochrome c into the cytosol [24]. This series of events activates the intrinsic apoptosis initiator caspase-9 and downstream effector caspases that are the same as the extrinsic pathway [24]. The B-cell lymphoma 2 (BCL-2) family of proteins regulate the intrinsic pathway at the level of the mitochondria [26]. Some of the BCL-2 family of proteins such as BCL-2 and myeloid leukemia 1 (MCL-1) are antiapoptotic, while others including BCL-2-associated X protein (BAX), BCL-2 antagonist or killer (BAK), and BCL-2 homology domain (BH3)-interacting domain death agonist (BID) are proapoptotic [24]. The antiapoptotic proteins play a role to inhibit proapoptotic proteins BAX/BAK by sequestering BH3-only proteins such as BID [27].

Many viruses can induce apoptosis through one or both of the apoptosis pathways. Receptors are crucial components to activate extrinsic pathway of apoptosis. Some virus families can increase apoptosis by increasing the expression of extrinsic pathway receptors such as Fas and their ligands on the cell surface. Epstein–Barr virus (EBV)-infected B cells express higher levels of the Fas receptor on their surface [28]. Both hepatitis B [29] and West Nile virus infections [30] increase the expression of death receptor–associated genes such as DR5 to activate death receptor–mediated apoptotic pathway. In addition, human immunodeficiency virus (HIV)-infected patients have reduced memory B cells by undergoing the extrinsic pathway of apoptosis involving TRAIL [31]. Vesicular stomatitis virus (VSV) can induce the intrinsic pathway of apoptosis when expressing wild-type viral matrix (M) protein or the extrinsic pathway of apoptosis when expressing a mutant version of the M protein [32]. In the subsequent sections, we have summarized the variety of stages of virus replication that leads to apoptosis.

#### 1.1 Apoptotic induction at the interface of virus–host receptor interaction

Apoptosis can be initiated when a virus particle binds to its receptor on the cell surface prior to initiation of viral genome replication. Measles virus activates the intrinsic pathway of apoptosis when the viral hemagglutinin binds to the cellular signaling lymphocytic activation molecule (SLAM) receptor [33]. Similarly, foot-and-mouth disease virus induces apoptosis via binding to the integrin receptor on immature dendritic cells [34].

#### 1.2 Apoptosis induction after sensing of viral nucleic acid replication intermediates

Many viruses activate apoptosis after starting genome replication in a susceptible cell. After uncoating, viral RNA genomes are detected by cytosolic RIG-I-like helicases (RLH) such as RIG-I, which typically activate IRF3 [35]. IRF3 is well characterized for its function as a transcription factor to stimulate the expression of IFN-mediated antiviral genes [35]. In some instances, IRF3 is also involved in the induction of apoptosis through a pathway commonly referred as the RLR-induced IRF3-mediated pathway of apoptosis (RIPA) [35]. Successful RIPA activation requires IRF3 to interact with BAX via its BH3 and to undergo translocation to the mitochondria, leading to the activation of apoptosis. This process requires additional proteins that are distinct from the antiviral transcription pathway [35]. Activation of the RIPA pathway is reliant on polyubiquitination of 2 specific lysine residues on the IRF3 protein [36]. Additionally, activation of IRF3 through RIG-I can also lead to the transcription of several proapoptotic proteins. Sendai virus, the Ankara strain of modified vaccinia virus (MVA), and reovirus rely on RIG-I-like helicases and IRF3 to activate apoptosis [37–39].

TLR3 is another RNA sensor that activates IRF3. TLR3 signaling and the associated signaling adaptor protein Toll/IL-1 receptor domain-containing adapter inducing IFN-beta (TRIF) can trigger apoptosis through the activation of caspase-8, which also requires receptor-interacting serine/threonine-protein kinase 1 (RIPK-1; [40,41]).

During DNA virus infection, the cytoplasmic DNA sensors cGas/STING can also mediate apoptosis induction. Infection with HSV-1 shows STING-dependent apoptosis at high doses [42]. During human T cell leukemia virus type 1 (HTLV-1) infection, inhibition of reverse transcription creates RNA intermediates that were sensed by STING triggering the IRF3-BAX complex and induction of apoptosis [43]. Without virus infection spontaneous activation of STING after gain of function mutation (N153S) can also activate ER stress and apoptosis [44].

#### 1.3 Apoptosis induction after viral-induced endoplasmic stress and unfolded protein response

Some viruses can impose ER stress through an increase in viral protein synthesis, which translates into the induction of the unfolded protein response (UPR) and, consequently, apoptosis [45]. Depending on the type of virus infection, 3 major UPR response pathways can be activated, namely, PKR-like ER kinase (PERK), inositol-requiring enzyme 1 (IRE1), and activating transcription factor 6 (ATF6) [45]. For example, upon infection with Zika virus, trophoblasts activate ER stress response and apoptosis [46]. West Nile virus induces the PERK pathway, which leads to the phosphorylation of eIF2ɑ and the activation of ATF4 [45]. ER stress activates ATF4 protein that subsequently stimulates the expression of the CCAAT-enhancer-binding protein homologous protein (CHOP) to initiate apoptosis [45]. Other viruses such as the Japanese encephalitis virus activate the PERK pathway, as the NS4B protein of Japanese encephalitis virus promotes dimerization of PERK [47]. Pseudorabies virus infection also activates the PERK-ATF4 and the IRE1-mediated apoptosis [48]. During avian reovirus infection, induction of the ATF6 pathway is also crucial for UPR-mediated apoptosis [49].

#### 1.4 Additional miscellaneous pathways leading to viral-mediated apoptosis induction

Viruses can also initiate or enhance infected cell apoptosis by altering cellular proteins and processes such as posttranslational modifications. Influenza virus can alter the functional activities of several host proteins to induce apoptosis. For example, the NS1 protein of influenza A virus (IAV) interacts with heat shock protein Hsp90 mediating the activation of caspase-9 and 3 [50]. Additionally, during influenza virus infection, the cellular protein BAD is phosphorylated to activate the intrinsic apoptosis pathway [51]. Influenza virus [52] and transmissible gastroenteritis virus [53] can activate apoptosis by facilitating the translocation of apoptosis-inducing factor (AIF) to the nucleus.

HIV is another example where a viral protein can induce apoptosis in a variety of ways. First, the Tat protein has been found to induce apoptosis by altering microtubule dynamics leading to the translocation of proapoptotic protein B cell lymphoma 2–interacting mediator of cell death (BIM) to the mitochondria, inducing mitochondrial membrane permeability and inhibiting cytochrome c oxidase [54,55]. In addition, the protease of HIV can cleave caspase-8 into Casp8p41, which engages BAX/BAK proteins to depolarize the mitochondria and activate caspase-9 [56].

The HPV18 E2 protein can trigger apoptosis by binding to the death effector domain of caspase-8, which induces caspase-8 activation after its oligomerization [57]. In HPV16, the E2 protein leads to the hyperactivation of caspase-8 by suppressing the function of the antiapoptotic protein c-FLIP making the infected cell hypersensitive to extrinsic apoptotic signaling [58]. Hepatitis C virus can initiate apoptosis through its NS4A and NS3A proteins that up-regulate BAX expression to initiate the intrinsic apoptosis [59].

### 2. Mechanisms of necroptosis induction during virus infection

**Fig 2:**
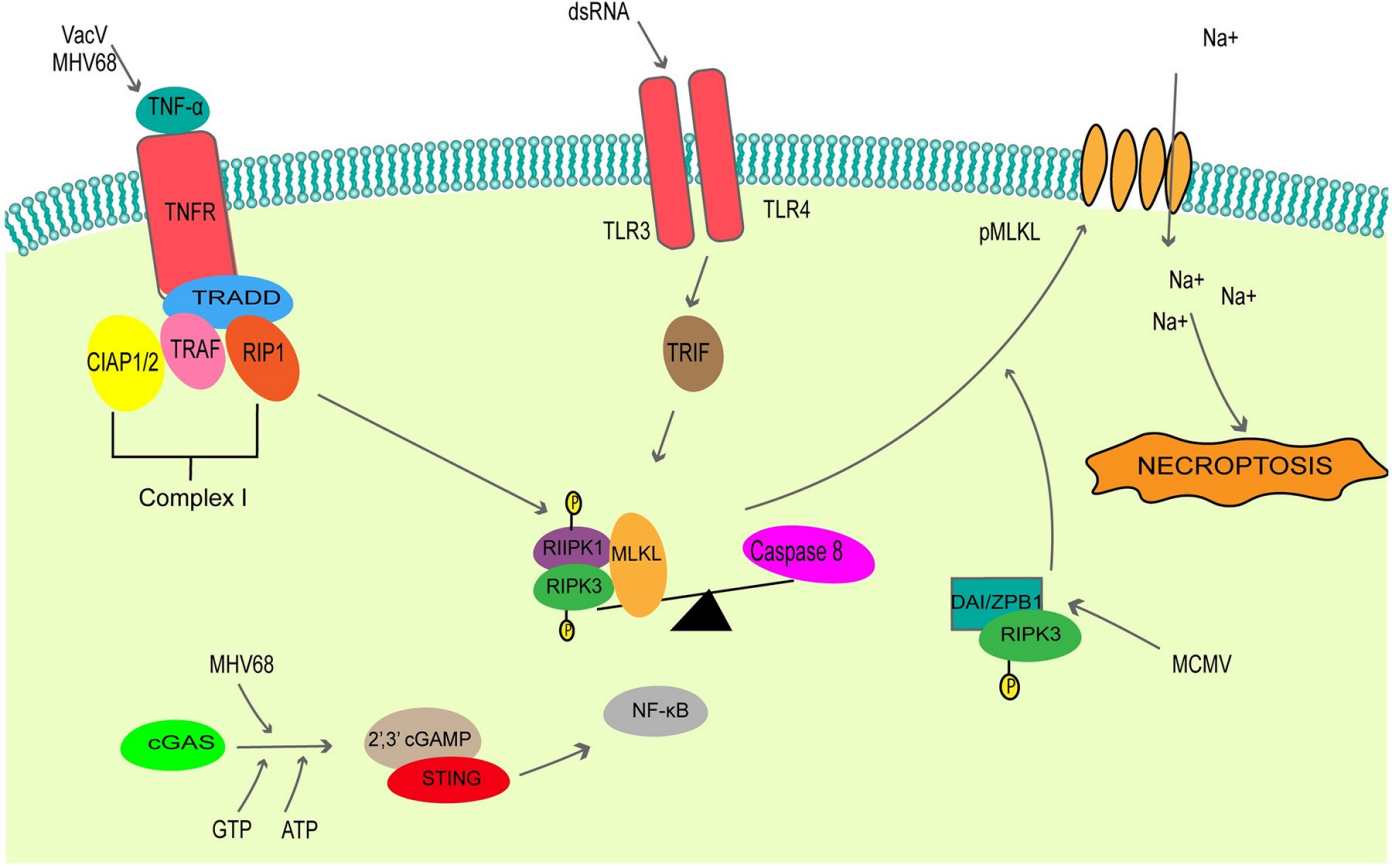
A summary of the major components of the necroptosis induction pathway following viral infection. Necroptosis is activated during viral infection through Toll-like receptor and TNF receptor activation, in addition to viral nucleic acid recognition by the intracellular sensors DAI/ZPB1. Necroptotic stimuli converge on the activation of the RIPK1/RIPK3 necrosome complex, in turn phosphorylating the effector protein MLKL to initiate cell lysis. cGAS, cyclic GMP–AMP synthetase; CIAP1/2, cellular inhibitor of apoptosis 1/2; DAI/ZPB1, DNA-dependent activator of IFN-regulatory factors/Z-DNA/RNA binding protein 1; dsRNA, double-stranded RNA; MCMV, murine cytomegalovirus; MLKL, mixed lineage kinase domain-like pseudokinase; NF-κB, nuclear factor-κB; pMLKL, phosphorylated MLKL; RIF, Toll/IL-1 receptor domain-containing adapter-inducing IFN-beta; RIP1, receptor-interacting protein 1; RIPK1, receptor-interacting serine/threonine-protein kinase 1; RIPK3, receptor-interacting serine/threonine-protein kinase 3; STING, stimulator of IFN genes; TLR3, Toll-like receptor 3; TLR4, Toll-like receptor 4; TNF, tumor necrosis factor; TNF-α, tumor necrosis factor alpha; TNFR, tumor necrosis factor receptor; TRADD, TNF-R-associated protein with death domain; TRAF, TNF-associated factor; 2′3′ cGAMP, 2′3′ cyclic GMP-AMP.

Necroptosis, a regulated form of necrosis, is often activated after engagement of Toll-like receptors (TLRs), death receptors, and the intracellular sensors Z-DNA/RNA binding protein 1 (ZBP1)/DAI [60,61]. Necroptosis is caspase independent and relies on TNF and Z-DNA/RNA to initiate the signaling cascade and kinases RIPK1/3 and mixed lineage kinase domain-like pseudokinase (MLKL) for execution (Fig 2). The morphological changes associated with necroptosis are distinct from apoptosis; these include organelle swelling and cell lysis [62]. In addition, during necroptosis, RIPK1/3 heterodimers form a complex that activates NF-kB-mediated proinflammatory response to allow the emission of cytokines/chemokines [63–65]. Furthermore, damage-associated molecular patterns (DAMPs), such as ATP and HMGB1, are also profusely secreted during necroptosis [66,67].

#### 2.1 Canonical (indirect) activation of necroptosis

The initiation of necroptosis through TNF, also known as the indirect necrosome formation and canonical pathway, leads to the formation of TNF-receptor-associated complex, which independently recruits receptor-interacting protein I (RIPK1) and TRADD through their death domains (DDs) [68]. The expression of TNF is regulated through various mechanisms, including virus infection. The detailed mechanisms for the activation of necroptosis after TNF stimulation is extensively reviewed elsewhere [65,69,70]. Vaccinia virus (VACV) [71] and murine gamma herpesvirus 68 (MHV68; [72]) infected cells utilize TNF-mediated necroptosis. The induction of necroptosis after MHV68 infection can also take place in a STING-dependent manner. The presence of cytosolic DNA in MHV68 [73] and VACV [74] infected cells can trigger the cGAS-STING pathway. cGAS interacts with cytosolic DNA to undergo an active site conformational change for the synthesis of cyclic GMP-AMP (cGAMP) from GTP and ATP [75,76]. cGAMP is a second messenger that binds and activates STING, leading to the activation of the NF-kB pathway, which stimulates TNF expression [77–80]. Secreted TNF binds to the TNFR to induce a signaling cascade that leads to an inflammatory response and necroptosis.

#### 2.2 Direct activation of necroptosis

Direct activation of necroptosis is initiated by the ZBP1/DAI sensors or TRIF adaptor protein [81]. Z-DNA and Z-RNA, nucleic acids with a left-handed conformation of the DNA and RNA structure, are found in actively transcribed regions of the genome and can be bound by Z-DNA binding proteins [82–85]. The Z-DNA binding proteins, upon interaction with Z-DNA, are thought to have roles in gene expression, DNA processing, tumor development, and viral pathogenicity [86–88]. ZBP1/DAI can recognize Z binding domains (ZBDs) on Z-DNAs and Z-RNAs in the cell [88].

During murine cytomegalovirus (MCMV) infection, induction of necroptosis required RNA synthesis but not viral replication since inhibition of RNA transcription but not of viral DNA replication prevented ZBP1-dependent necroptosis [89]. In addition, the binding of ZBDs to ZBP1/DAI are required to induce necroptosis [89]. During influenza A infection, the Z-RNAs generated during virus replication are sensed by ZBP1/DAI [90]. Sensing of Z-RNA leads to dimerization of ZBP1/DAI to interact with RIPK3 via its RHIM domain to initiate necroptosis [91,92].

TLR are activated by several pathogen stimuli to drive a variety of PCD. TLR3 and TLR4 signal through an adaptor protein TRIF when stimulated by either dsRNA [93]. When caspase-8 is inhibited during this stimulation, TLR3 and TLR4 activate necroptosis through TRIF [61]. TRIF is a RHIM-containing protein that forms a noncanonical necrosome by activating RIPK3 [61].

#### 2.3. Execution of necroptosis downstream of RIPK3 activation

Most of the necroptotic pathways converge at RIPK3 recruitment, phosphorylation, and activation. RIPK1 is required for RIPK3 auto-phosphorylation and cross-phosphorylation during indirect canonical necrosome formation [94]. In the noncanonical pathway, the interaction of RIPK3 with DAI or TRIF causes RIPK3 activation and auto-phosphorylation [94]. Activated RIPK3 phosphorylates MLKL, inducing a conformational change that exposes 4 helical bundle domains, which causes oligomerization [95]. MLKL is hypothesized to act in 2 ways. It is suggested that phosphorylated MLKL (P-MLKL) translocates to the plasma membrane via interactions with the amino-terminal of phosphatidylinositol (PIP) and induces membrane rupture [96]. It is also speculated that P-MLKL associates with cation channels, leading to ion influx [97].

### 3. Mechanisms of pyroptosis induction during virus infection

**Fig 3:**
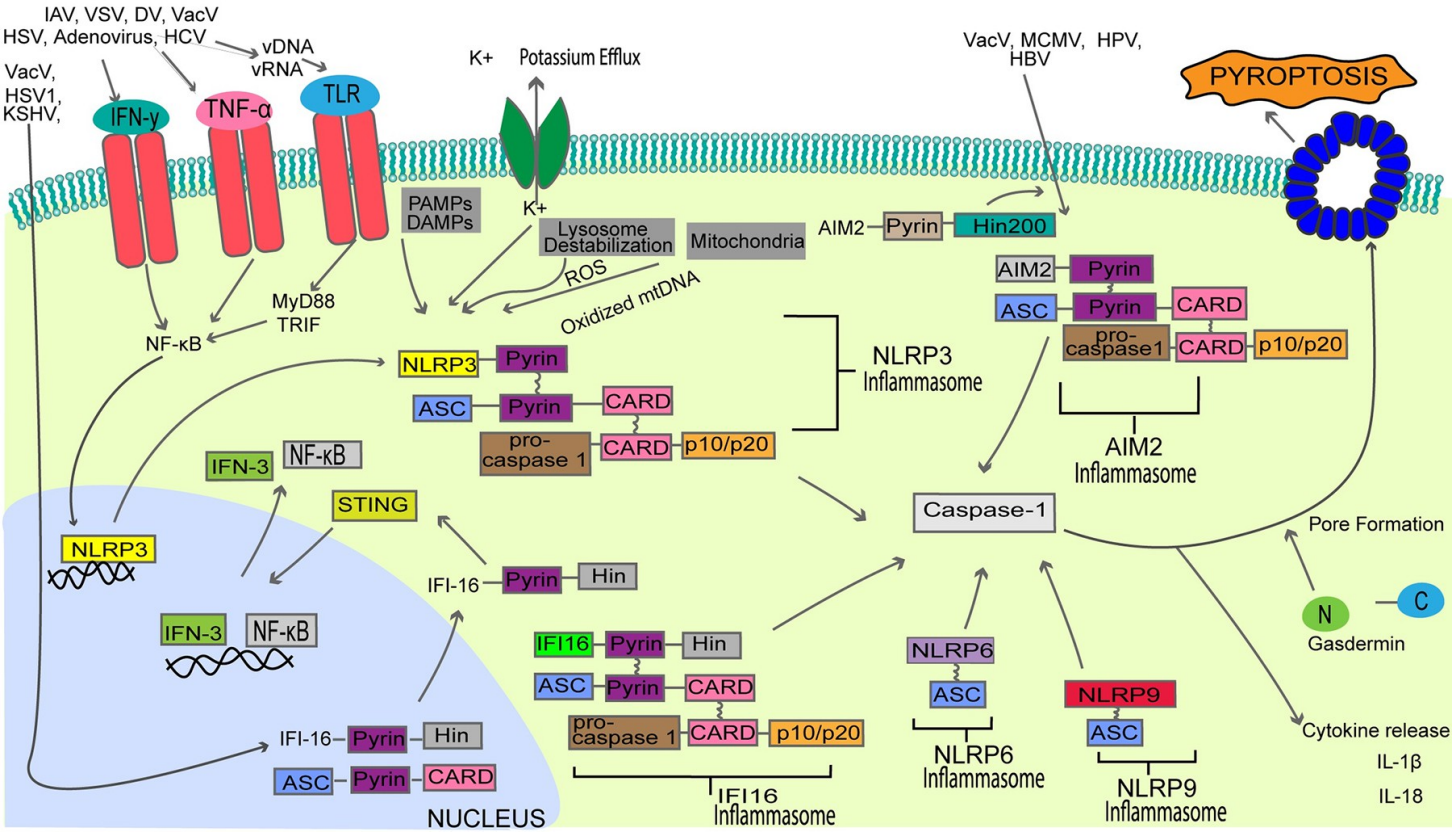
A summary of the major components of the pyroptosis induction pathway following viral infection. Viral nucleic acids can stimulate pyroptosis through the activation of the NLRP3/6/9 inflammasome, AIM2 inflammasome, and IFI16 inflammasome. Inflammasome activation stimulates the recruitment and activation of pro-caspase-1, resulting in the downstream cleavage of gasdermin proteins into C-terminal and N-terminal portions. The N-terminal gasdermin portion catalyzes pore formation and proinflammatory cytokine release. AIM2, absent in melanoma 2; ASC, Apoptosis-associated speck-like protein containing a CARD; CARD, caspase activation and recruitment domain; DAMP, damage-associated molecular pattern; DV, dengue virus; HBV, hepatitis B virus; HCV, hepatitis c virus; HPV, human papillomavirus; HSV, herpes simplex virus; IAV, influenza A virus; IFI16, IFN-gamma-inducible protein 16; IFN-3, interferon-3; IFN-y, interferon gamma; KSHV, Kaposi sarcoma–associated herpesvirus; MCMV, murine cytomegalovirus; mtDNA, mitochondrial DNA; NF-κB, nuclear factor-κB; NLRP3, Nod-like receptor family pyrin domain containing 3; PAMP, pathogen-associated molecular pattern; ROS, reactive oxygen species; STING, stimulator of IFN genes; TLR, Toll-like receptor; TNF-α, tumor necrosis factor alpha; TRIF, Toll/IL-1 receptor domain-containing adapter inducing IFN-beta; VACV, vaccinia virus; VSV, vesicular stomatitis virus.

Pyroptosis plays a crucial role in regulating host immunity against viruses. It is a form of inflammatory cell death that can be initiated after recognition of conserved PAMPs by host pathogen recognition receptors (PRRs) (Fig 3). The molecular response of host cells to pyroptosis is characterized by pore formation, plasma membrane rupture, and the activation and release of proinflammatory cytokines.

A critical step in pyroptosis activation is gasdermin cleavage [98]. Gasdermin proteins are cleaved by caspases into N- and C-terminal fragments. The N-terminal portion of gasdermin functions as the pore-forming domain by inserting itself into the lipid bilayer of the cytoplasm and oligomerizing to form pores in the cell membrane [99]. Pore formation induces osmotic imbalance and ultimately results in cell lysis [99,100]. Like necroptosis, pyroptosis results in the release of a variety of danger molecules such as ATP, HMGB1, and cytokines including IL-18 and IL-1β [101–103].

Overall, pyroptosis is initiated through 2 distinct pathways: the canonical pathway and the noncanonical pathway. The canonical pathway of pyroptosis, otherwise known as caspase-1-dependent PCD, is commonly activated by several viruses. In contrast, the noncanonical pathway that results in the activation of caspase-4, caspase-5, and caspase-11, after sensing of bacterial PAMPs, thus will not be discussed in this review.

### 3.1. The canonical pathway of pyroptosis activation

The canonical inflammasome sensors are required to induce caspase-1-dependent pyroptosis. Various canonical inflammasomes mediate virus-induced pyroptosis, including the Nod-like receptor family pyrin domain containing 3 (NLRP3), NLRP6, and NLRP9 inflammasomes, AIM2 inflammasome, and the IFN-gamma-inducible protein-16 (IFI16) inflammasome [10,11,104]. Procaspase-1 is a component of the inactive inflammasome with 3 main domains, including 2 catalytic subunits, p10 and p20, and a noncatalytic caspase activation and recruitment domain (CARD). Activation of the inflammasome containing procaspase-1 is required for cleavage of the inactive caspase zymogen [105].

#### 3.1.1. NLRP3/6/9 inflammasome-mediated viral pyroptosis

Many RNA viruses (IAV, VSV, hepatitis C virus, dengue virus (DV)) and some DNA viruses (herpes virus, adenovirus, VACV) are sensed through NLRP3. Unlike AIM2 and IFI16, activation of the NLRP3 inflammasome requires 2 distinct signals. The priming signal is the first one often generated after NF-κB production, which is activated when a variety of ligands stimulate the PRRs (e.g., TLR), TNFR, RIG-I, and IL-1R [106–108]. This up-regulates the transcription of NLRP3 and pro-IL-1β, as well as posttranslational modifications such as NLRP3 phosphorylation and ubiquitination [106,109–112]. Examples of pathways that stimulate NF-kB include IFN-γ and TNF-α [113,114]; MyD88 and TRIF mediated response downstream of TLR stimulation [106], activation of RIG-I-mediated responses after cytosolic viral RNA detection [115].

A second signal is required for the pyrin domain of NLRP3 to bind ASC, causing ASC oligomerization and ASC speck formation [116]. The internal CARD and activation domain recruits and activates caspase-1 [117]. Caspase-1 activation leads to the downstream cleavage of N-terminal gasdermin and pore formation. The exact mechanism for the execution of the second signal is not known.

Two relatively new inflammasomes NLRP6 and NLRP9 can also activate pyroptosis. Activated NLRP6 along with ASC form an inflammasome to activate caspase-1 and caspase-11 [10,11,104]. Furthermore, NLRP9b is one of the 3 mouse paralogs of NLRP9 and recognizes short dsRNA stretches in concert with the RNA helicase Dhx9 to form inflammasome complexes with the adaptor protein ASC to activate caspase-1-mediated gasdermin D (Gsdmd)-induced pyroptosis and the maturation of IL-18 [118–120].

#### 3.1.2. AIM2 inflammasome-mediated viral pyroptosis

The AIM2 inflammasome, a member of the ALR family, consists of a hematopoietic IFN-inducible nuclear (HIN)-200 domain that predominantly binds to a wide range of cytosolic double-stranded (ds)-DNA and, in rare cases, cytosolic RNA [121,122]. Under normal states, the inflammasome maintains an autoinhibited state. However, the interaction of the sugar-phosphate backbone of dsDNA with the HIN-200 domain functionally displaces the pyrin domain from the inhibited state, liberating the pyrin domain to interact with the pyrin domain of ASC [122,123]. The CARD domain of procaspase-1 is recruited through the CARD domain of ASC, allowing for inflammasome assembly and caspase-1 activation. The exact mechanism by which viral DNA is exposed for AIM2 sensing is not precisely known, although several viral dsDNA ligands of VACV, human papillomavirus (HPV), hepatitis B virus, and mouse cytomegalovirus (MCMV) are sensed in an AIM2-dependent manner [124,125].

#### 3.1.3. IFI16 inflammasome-mediated viral pyroptosis

Closely associated with the AIM2 inflammasome is the IFI16 inflammasome, a canonical inflammasome with an ability to bind single and double-stranded DNA, triggering pyroptosis during viral infection. Initially, IFI16 was discovered as a viral DNA sensor of HSV-1, VACV, Kaposi sarcoma–associated herpesvirus (KSHV), and single-stranded DNA from HIV-infected CD4+ T cells [126]. IFI16 is mainly localized within the nucleus; however, it is also known to translocate to the cytoplasm to engage in antiviral immune response [126,127]. As a regulator of pyroptosis, cytoplasmic IFI16 can colocalize with ASC via its PYD domain after viral DNA recognition by the HIN domain [128]. Such interactions allow for caspase-1 activation, leading to cytokine release and pyroptosis.

During its latent period, KSHV, a dsDNA virus, resides within the nucleus of infected cells, tethered to host cell chromatin. During KSHV infection, oligomers of IFI16 can be seen to colocalize in the nucleus of infected endothelial cells with the KSHV genome in a nonspecific way; this cumulative interaction launches inflammasome formation, which recruits caspase-1 and ASC, inducing pyroptotic cell death and mature inflammatory cytokine release [129]. It is most probable that KSHV binding to IFI16 results in structural changes that allow its interaction with ASC [130].

### 4. Mechanisms of ferroptosis activation during virus infection

Ferroptosis is a type of PCD that is initiated after sensing free iron accumulation and increased lipid peroxidation. These two are coupled to or caused by the accumulation of intracellular reactive oxygen species (ROS) [131,132]. Lipid peroxidation refers to the result of oxidative attack on polyunsaturated fatty acids by ROS. One of the ways that ferroptosis is induced is via the inhibition of the system Xc^−^ cystine/glutamate antiporter, which is a heterodimer made of solute carrier (SLC) family 7 member (SLC7A11) and SLC3A2. This antiporter functions to import cystine into the cell where it is reduced to cysteine and used for glutathione (GSH) synthesis [133]. GSH works to reduce ROS in the cell via the function of GSH peroxidases such as glutathione peroxidase 4 (GPX4). Many molecules that induce ferroptosis, such as erastin, induce ferroptosis by inhibiting system Xc^−^ or modulating the pathway in some way [132]. Morphologically, ferroptosis mainly manifests as shrinkage of mitochondria with increased membrane density and reduction in or vanishing of mitochondrial cristae, which is a different process from other modes of cell death [132].

Studies elucidating the mechanisms of ferroptosis induction in virus-infected cells are very limited. One study demonstrated that Newcastle disease virus (NDV) can induce ferroptosis [134]. Cells infected by NDV demonstrated an increase in ROS as well as lipid peroxides. The mechanism by which NDV induces ferroptosis is by inhibiting the Xc-GSH-GPX4 pathway and appears to require the p53 protein [134]. Another study highlighted that the expression of the hepatitis A virus 3C protease induced ferroptosis [135]. There is a direct link between the DNA sensor STING and ferroptosis. The classic ferroptosis inducer erastin induces the accumulation of STING in the mitochondria to ROS production and lipid oxidation [136]. Both of these are hallmarks of ferroptosis, and depleting STING1 reduced sensitivity to ferroptosis [136]. In another study, zalcitabine-induced mitochondrial DNA stress activates STING-mediated DNA sensing pathway leading to autophagy dependent ferroptotic cell death via lipid peroxidation, independent of the IFN response [137]. These studies highlight the need for future studies to determine how other viruses activate and inhibit ferroptosis.

### 5. Mechanisms of paraptosis activation during virus infection

Paraptosis is a type of PCD characterized by cytoplasmic vacuolation, usually involving the swelling of the ER and mitochondria. Paraptosis is often accompanied by an alteration of Ca^2+^ and redox homeostasis [138], as well as by accumulation of misfolded proteins causing ER stress and cell death [139]. However, these features are not always present in cells undergoing paraptosis. In most cases, paraptosis depends on mitogen-activated protein kinase (MAPK) family members, such as c-Jun N-terminal protein kinase 1 (JNK1), p38, and mitogen-activated protein kinase kinase 2 (MEK-2), and it can be inhibited by the multifunctional adapter protein AIP-1/Alix.

Similar to ferroptosis, there is little research on virus-induced paraptosis. Zika virus infection can activate ER-dependent, large, cytoplasmic vacuoles characteristic of paraptosis. The ability of the virus to create these paraptotic vacuoles was dependent on its ability to replicate within ER-derived membranes [140]. In another study, Singapore grouper iridovirus (SGIV) induced STAT-3-dependent paraptosis of fish cells [141]. Further research is required to understand mechanisms of virus-induced paraptosis.

### Inhibition of programmed cell death by viruses

Depending on the type of virus, infection cells attempt to abort the infection by activating PCD. On the other hand, viruses highjack a variety of cellular processes including the initiation and execution of PCD starting early after infection. For example, herpesviruses and poxviruses, which utilize over one-third of their genome to counteract the host antiviral response [142]. In this section, we summarize the various strategies viruses deploy to inhibit apoptosis, necroptosis, and pyroptosis (Tables 1 and 2).

**Table 1:** Mechanisms of apoptosis inhibition by viruses.

PCD	Family	Virus	Viral Protein	Proteins Role in Inhibition
**Apoptosis**	*Poxviridae*	VACV	F1L [145,204]	BCL-2 homolog, sequesters BIM
			N1L [205]	BCL-2 homolog, NF-kB inhibition
			A46 [206]	
			A49 [207,208]	
			A52 [209]	
			B14 [209]	
			K7 [210]	
			SPI-1/SPI-2/SPI-3 [211–215]	Caspase inhibition
			CrmB/CrmC/CrmE [216]	Mimics TNFR1/2
			E3 [217]	Inhibits activation of PKR
			D9/D10 [218]	
			M1L [219]	Inhibits apoptosome functions and blocks caspase-9 processing
		VARV	VAR F1L [220]	BCL-2 homolog, sequesters BID, BAK, and BAX
			CrmB [221]	TNFR ½ mimic
		CPXV	CrmA [146,147]	Caspase-8 inhibition
			CrmB [222]	TNFR1/2 mimic
			CrmC [222]	
			CrmD [222]	
			CrmE [156]	
			vCD30 [223]	
		ECTV	EMV025 [224]	BCL-2 homolog, sequesters BAK to inhibit BAK and BAX activity
			vCD30 [225]	TNFR1/2 mimic
		MYXV	M11L [226]	BCL-2 homolog, sequesters BAK and BAX
			SERP1 [227]/SERP2 [228]/SERP3 [229]	Caspase inhibition
			M-T2 [230]	Mimics TNFR1/2
			M131 [231]	SOD homolog
			M029 [232]	Inhibits activation of PKR
		SFV	T2 [230,233]	TNFR1/2 mimic
			S131 [231]	SOD homolog
			SPV032 [217]	Inhibits PKR activation
		TANV	16L [234]	BCL-2 homolog
			2L [235]	TNFR1/2 mimic
		ORFV	ORFV125 [236–239]	BCL-2 homolog
		SPPV	SPPV14 [239,240]	BCL-2 homolog
		DPV	DPV022 [241]	BCL-2 homolog
		FPV	FPV039 [242,243]	BCL-2 homolog, sequester all BH3-only proteins
		CNP	CNP058 [244]	BCL-2 homolog
		MCV	MC163 [245]	SOD homolog
			MC159 [246–250]	- Inhibitor of TNF-α/FasL induced apoptosis - cFLIP homolog, binds FADD - BCL-2 homolog
			MC160 [246,247,251]	- Inhibitor of TNF-α/ induces NF-kB prosurvival signaling - cFLIP homolog, binds caspase-8
	*Herpesviridae*	HVT	vNr-13 [252]	BCL-2 homolog
		HVS	HVS-Bcl-2 [253]	BCL-2 homolog
			ORF-71 [249]	c-FLIP and BCL-2 homolog
		HSV-1	ICP22 [163,254–257]	Caspase-3 inhibition, antagonizes p53
			US3 [164,258–261]	Blocks cleavage of procaspase-3 - activates PI3-K/akt pathways, - inhibits NF-kB pathway by hyper-phosphorylating p65, - decreases expression of IL-8, - phosphorylates proapoptotic members of BCL-2 family: BAD and BID
		HSV	US5 (glycoprotein J) [262,263]	Suppresses caspase-3 and 8 activation, inhibits F_o_F_1_ ATP synthase function and ROS formation to suppress apoptosis
		HSV-1	US6 (glycoprotein D) [264–267]	Inhibits Fas-mediated apoptosis by activating NF-kB pathway
		HSV	US8 (glycoprotein E) [268]	Degrades BIM protein
		HSV 1/2	ICP6/ICP10 [269]	Inhibits caspase-8 to prevent TNF-induced apoptosis
		MCMV	M36 [270]	Interacts with and inhibits caspase-8
		HCMV	UL36 [271]	Interacts with and inhibits caspase-8
		MHV-68	M11 [272]	Neutralizes proapoptotic BCL-2 family of proteins Inhibits Beclin1-dependent autophagy
		EHV-2	E8 [249]	c-FLIP homolog, inhibits procaspase-8 activation and Bcl-2 homolog
		HHV-8/KSHV	ORF-71 [249,273–275]	c-FLIP homolog, inhibits CD95 mediated apoptosis and BCL-2 homolog
			K13 [246]	c-FLIP homolog and activates NF-kB
		BHV-4	ORF-16 [249,276]	c-FLIP homolog, prevents activation of caspase-8 and BCL-2 homolog
	*Adenoviridae*	E1B 19K	E1B 19K protein [277,278]	Bcl-2 homolog and blocks p53 meditated apoptosis
	*Baculoviridae*	OpMNPV	IAP3 [155]	IAP3 homolog
		*Anticarsia gemmatalis* MNPV	IAP3 [155]	IAP3 homolog
		*Helicoverpa armigera* NPV	IAP3 [155]	IAP3 homolog
		AmEPV	IAP3 [155]	Carries p35 and IAP3 homolog
			AMViap [279]	Caspase-3 and 9 inhibitor
		CpGV	IAP6 [155]	IAP6 homolog
		*Pieris rapae* GV	IAP6 [155]	IAP6 homolog
		*Cryptophlebia leucotreta* GV	IAP6 [155]	IAP6 homolog
		*Phthorimaea operculella* GV	IAP6 [155]	IAP6 homolog
		*Hyphantria cunea* NPV	IAP3 [155]	IAP3 homolog
		AcMNPV	IAP1 [155]	Carries p35 and IAP1 homolog

AcMNPV, *Autographa californica* multiple nucleopolyhedrovirus; AmEPV, *Amsacta moorei* entomopoxvirus; ASFV, African swine fever virus; BIR, Baculovirus IAP repeat; CMV, cytomegalovirus; CpGV, *Cydia pomonella* granulovirus; EHV, equine herpes virus; EV71, enterovirus 71; HHV, human herpes virus; HPIV, human parainfluenza virus; HVS, Herpes virus Saimiri; HVT, herpesvirus of turkeys; IAP, inhibitor of apoptosis protein; ICP, infected cell protein; KSHV, Kaposi sarcoma–associated virus; LAT, latency-associated transcript; MHV-68, murine gammaherpesvirus-68; MV, measles virus; MYXV, myxoma virus; NiV, Nipah virus; OpMNPV, *Orgyia pseudotsugata* MNPV; PEDV, porcine epidemic diarrhea virus; R1, ribonucleotide reductase; SeV, Sendai virus; SFV, Shope fibroma virus; SVV, Seneca Valley virus; v-FLIP, viral FLICE-like inhibitory proteins; VZC, varicella zoster virus.

**Table 2:** Mechanisms of necroptosis and pyroptosis inhibition during virus infection.

PCD	Family	Virus	Viral Protein	Proteins Role in Inhibition
**Necroptosis**	*Herpesviridae*	MCMV	M45 [169,280]	RHIM-dependent amyloid plague formation leading to sequestering of RIPK1, RIPK3, and DAI
		HSV1	ICP6 [170]	
		HSV2	ICP10 [269]	
		HCMV	UL36 [281,282]	Targeting of MLKL for proteasomal degradation
		HSV1	ICP6 [169]	IPAM-dependent aggregation and autophagy of RIPK1
		MCMV	M45 [169]	
		EBV	LMP1	Ubiquitination of RIPK1 causing prosurvival signaling [175]
				Deubiquitination of RIPK3 preventing necrosome formation [175]
				Hypermethylation of RIPK3 promoter [175]
	*Poxviridae*	CPXV	vIRD [176]	RIPK3 ubiquitination and proteasomal degradation
		VARV		
		MPXV		
		ECTV		
		*Suipoxvirus*	E3 [283,284]	Sequestering z-RNA inhibiting PAMP detection
		*Caprpoxvirus*		
		VARV		
		VACV		
		*Carpripoxvirus*	vMLKL [284,285]	Sequestering RIPK3 and RIPK1 while lacking the ability to insert into the cell membrane
		*Suipoxvirus*		
		*Avipoxvirus*		
		*Leporipoxvirus*		
	*Orthomyxoviridae*	IAV	Hemagglutinin of pandemic IAV strains [286]	Inhibition of RIPK3-mediated necroptosis
**Pyroptosis**	*Coronavidae*	SARS-Cov-2	NSP5	Cleavage of Gasdermin D into N-terminal lacking critical residues [182]
		MARS-Cov		
		PEDV	NSP4	Cleavage of Gasdermin D into N-terminal lacking critical residues [287]
		TGEV		
		SARS-Cov-2	NP [288]	Blockage of Gasdermin D cleavage by caspase-1
	*Picoronavidae*	EV71	3Cpro [289,290]	Cleavage of Gasdermin D into N-terminal lacking critical residues
		SVV		
	*Arterviridae*	EAV	NSP4 [287]	Cleavage of Gasdermin D into N-terminal lacking critical residues
	*Asfarviridae*	ASFV	P5273R [287]	
Unconfirmed Pyroptosis	*Herpesviridae*	EBV	miRNA-BART15 [291]	NLRP3 inflammasome formation
		KSHV	Orf63 [292]	NLRP1 inflammasome formation
	*Orthomyxoviridae*	IAV	NS1 [293]	NLRP3-ASC inflammasome interaction
	*Paramyxoviridae*	MV	Protein V [294]	Localizes NLRP3 to peri-nuclear space
		SeV	Protein V	NLRP3 Inflammasome-ASC formation [292]
		NiV		NLRP3 Inflammasome-ASC formation [292]
		HPIV		NLRP3 Inflammasome-ASC formation [292]
	*Papillomaviridae*	HPV	E7	Ubiquitination and degradation of IFI16 inflammasome [295]
	*Poxiviridae*	SFV	PYD-only Protein	Inflammasome formation [292]
		MYXV	M13L [292]	Inflammasome formation

ASFV, African swine fever virus; EV71, enterovirus 71; HPIV, human parainfluenza virus; MV, measles virus; MYXV, myxoma virus; NiV, Nipah virus; PEDV, porcine epidemic diarrhea virus; SeV, Sendai virus; SFV, Shope fibroma virus; SVV, Seneca Valley virus.

#### 1. Viral-mediated inhibition of apoptosis

Viral BCL-2 (vBCL-2) homologs are among the best studied viral inhibitors of apoptosis. vBCL-2 homologs, widely distributed throughout *Poxviridae*, *Herpesviridae*, *Asfarviridae*, and *Iridovirdae*, unilaterally possess a hydrophobic BH3 binding domain capable of apoptosis inhibition. Broadly, vBCL-2 homologs either directly inhibit BAX and BAD oligomerization, preventing mitochondrial pore formation and cytochrome c release, or sequester proapoptotic BCL-2 proteins, diminishing proapoptosis signals [143,144]. Although functionally analogous, vBCL-2 homologs interact with diverse and distinct proapoptotic BCL-2 proteins. Within *Poxviridae*, VACV encodes F1L, which adopts a unique domain swapped BCL-2 topology forming ligand-specific interactions with either BAX or BAK to directly inhibit oligomerization and mitochondrial pore formation, or with BIM, sequestering the proapoptotic signal [145].

Viruses can also block apoptosis by inhibiting a variety of caspases. Expressed during cowpox virus (CPXV) infection, CrmA was the first serpin-like protein found to potently inhibit caspase-8, caspase-1, and caspase-10 [146,147]. Homologs of CrmA in *Orthopoxvirus* and C*horodopoxvirus* display a broad spectrum of caspase inhibition through a pseudo-substrate mechanism [148,149]. Furthermore, viruses can also specifically inhibit caspase-8. Viral inhibitors of caspase-8 (vICA) are distributed throughout *Herpesviridae*. ICP6 of HSV1 (ICP10 in HSV2), UL36 in human, and M36 in MCMV directly interact with the death effector domains (DEDs) on procaspase-8 inhibiting FADD association and procaspase-8 cleavage, protecting against extrinsic apoptosis [150–152]. Additionally, members of KSHV encode cellular FLIP analogs, termed viral FLIPs (vFLIPs). vFLIPs resemble the short isoform of cellular FLIPs and again completely block caspase-8 proteolytic activity [153,154]. Moreover, large DNA viruses infecting arthropods encode viral inhibitors of apoptosis proteins (vIAPs) containing a baculoviruses IAP repeat (BIR) motifs that binds and inhibit apoptosis mediators Hid, Reaper, or Grim. Moreover, really interesting new gene (RING) domains on vIAPs facilitate the ubiquitination and degradation of these proapoptotic proteins, reinforcing their antiapoptotic effects [155].

Outside of the largely conserved mechanisms, virus family-specific mechanisms are extremely diverse. Poxviruses encode TNFR homologs (vTNFRs) capable of binding and depleting proapoptotic TNF along with other immune-modulatory cytokines [156–158]. Additionally, VACV encodes the E3 protein to prevent PKR activation by binding dsRNA [159]. Viral Golgi antiapoptotic protein (vGAAP) of camelpox virus and some strains of VACV have also been shown to inhibit intrinsic apoptosis through a Ca^2+^-dependent mechanism [160,161]. Varster zooster virus (VZV) of *Herpesviridae* encodes IE63 capable of inhibiting apoptosis through inhibition of eIF-2α [162,163]. Finally, α-herpesviruses encode US3, adept at phosphorylating BAD and caspase-3, preventing activation [164,165]. Overall, large DNA viruses precisely target apoptosis through distinct and comprehensive mechanisms.

### 2. Viral-mediated inhibition of necroptosis

Viruses inhibit necroptosis mostly at the level of RIPK1/3 and MLKL. In herpesviruses and poxviruses, inhibiting the kinases RIPK1/3 is a very common process. Both viruses encode various inhibitors of RHIM-dependent functions of RIPK1/3. Multiple herpesviruses encode RHIM-containing proteins that inhibit the formation of amyloid-like filamentous necroptotic signaling complexes; effectively inhibiting the RIPK1-RIPK3 signaling axis and MLKL activation [64,166–168]. The first identified RHIM-dependent inhibitor was the viral ribonuclease reductases subunit (R1) homolog, M45 of murine cytomegalovirus (MCMV) [167,168]. M45 forms homo amyloid-like filamentous structures that bind RHIM domains of RIPK1, RIPK3, and ZBP1/DAI, effectively sequestering RHIM containing necroptotic machinery, inhibiting MLKL activation and cell lysis [167,168]. Additionally, viral UL39 R1 homologs of HSV1 and HSV2, termed ICP6 and ICP10, respectively, form similar RHIM-dependent amyloid-like fibrils to make the kinases dysfunctional [169]. However, underlying mechanisms of dysfunctional amyloid fibril formation differ between HSV and MCMV [170,171]. Furthermore, the RHIM-dependent inhibitor analog of Varicella zoster virus (VZV) ORF20 imposes similar amyloid-like fibrils [172].

Viruses also often facilitate the degradation of RIPK1 and RIPK3. Multiple human herpesviruses, such as herpes simplex virus (HSV), EBV, and KSHV, encode homologs of MCMV M45 protein. These viral inhibitors contain induced protein aggregation motifs (IPAMs) that facilitate insoluble protein aggregation of RIPK1, directing its degradation [173]. IPAM sequences are widely homologous in over 70 herpesviruses, baculoviruses, and giant viruses; however, functional conservation of this mechanism is unconfirmed [173]. EBV also employs LMP1 to manipulate ubiquitination of RIPK1 and RIPK3. LMP1 directly associates with the activation domain of RIPK1 promoting ubiquitination through the E3 ligase TRAF2, restricting RIPK1 to the plasma membrane where it maintains prosurvival signaling [174]. Concurrently, LMP1 facilitates deubiquitylation of RIPK3, preventing necrosome formation [174]. Finally, LMP1 exerts epigenetic control of RIPK3 through 2 axes: LMP1 up-regulates DNMT1 to directly methylate the transcription start site (TSS) of RIPK3 and also down-regulates ten-eleven translocation methylcytosine dioxygenase (TETs) to further methylate promoters of RIPK3 [175].

Poxviruses also target RIPK3 through viral inducers of RIPK3 degradation (vIRD). vIRD analogs are found in CPXV, variola virus (VARV), monkeypox virus (MPXV), and Ectromelia virus (ECTV) and directly bind to RIPK3 through their Ankyrin repeats, while their F-box domains link to host SKP1-Cullin1-F-box machinery. This interaction stimulates ubiquitination of RIPK3 and subsequent proteasomal degradation [176]. Interestingly, VACV, an attenuated virus used to eradicate VARV, lacks a functional vIRD homolog potentially shedding light on its benign and immunogenic nature [176].

Multiple *Poxviridae* inhibit necroptosis by inhibiting MLKL. C*hordopoxvirinae* encode E3, a viral protein possessing a z-RNA binding domain. Z-RNA, produced during viral replication, binds to the N-terminal Zα domain of E3, effectively sequestering the viral PAMP inhibiting ZBP1/DAI-dependent necroptosis [177,178]. *Suipoxvirus*, *Carpirpoxvirus*, *Avipoxvirus*, and *Lepropoxvirus* families also express viral MLKL (vMLKL) analogs [178,179]. vMLKLs retain high homology of the pseudokinase domain, allowing dominant sequestration of RIPK3 from RIPK1; however, lack the 4 helical bundle domains essential for membrane insertion and cell lysis. Human cytomegalovirus (HCMV) also ubiquinates MLKL through the viral protein UL36, causing necroptotic inhibition in human but not murine cell lines [180,181].

In summary, the large diversity and redundancies in viral inhibition of necroptosis support the emerging theory that necroptosis and viruses have coevolved specific virus–host mechanisms to antagonize the other, further highlighting importance of necroptosis in antiviral immunity and viral replication.

### 3. Viral-mediated inhibition of pyroptosis

Overall, the mechanisms by which viruses inhibit pyroptosis remain largely unknown. However, plus sense RNA viruses, such as *Coronaviridae*, utilize viral proteases to inhibit pyroptosis [182–184] (Table 2). Highly conserved viral proteases, such as NSP5 in coronaviruses, directly bind and cleave Gasdermin D in porcine and human cells, producing a truncated N-terminal Gasdermin D, lacking essential residues from caspase-1 cleavage [98]. Interestingly, a singular large dsDNA virus, African swine fever virus, has shown a similar mechanism through the viral protease S273R [185].

Additionally, within the *Coronaviridae* family, SARS-CoV-2 nucleocapsid protein (NP) blocks caspase-1-dependent cleavage of Gasdermin D through unidentified mechanism [186]. Upstream of terminal events, multiple viruses have been implicated in inhibiting PRR interactions, inflammasome formation, and ILb release, these events are summarized in Table 2; however, inhibition of pyroptosis has not been directly confirmed [187–192].

### Perspectives and future studies

The complexity of viral sensing pathways, diversity of animal viruses, and the ability of viruses to modulate PCD using their accessory proteins has been challenging to build a universal framework to dissect how viral ligand sensing interacts with the PCD machinery. Most studies we reviewed use live virus infections to study PCD. Hence, it is largely unknown how viral genome sensing in the context of inactivated and replication-defective virus stimulation regulates the PCD machinery. Many viral nucleic acid–like ligands including synthetic dsRNA:Poly I:C [193] and cGAMP [194] can activate cell death, suggesting the likelihood that viral genomes may also directly activate PCD, although similar studies are limited. In relation to this, it remains unknown if viral RNA and DNA genomes engage distinct PCD pathways.

An outcome of nucleic acid sensing is establishment of antiviral state via expression of various ISGs that exert direct antiviral effects in many ways. Although some ISGs have been extensively studied [195], the modes of action of a large majority of ISGs are still unknown [196]. There are more than 15 putative ISGs with proapoptotic functions, although their direct effect on PCD regulation is not known [197–199]. Furthermore, a large-scale proteomics study identified more than 200 novel PCD-associated proteins directly interact with some of the 104 inducibly expressed ISGs [196]. In support of this, IFN-induced transmembrane proteins 3 (IFITM3) directly regulates PCD induction in Zika virus–infected cells [140,200] and overexpression of ISG54 [201] and ISG12b2 [202], independent of IFN stimulation, activate apoptosis. The broader relevance of ISG modulating PCDs in the context of viral infection remains to be investigated.

Much is unknown how PCD influences viral pathogenesis. On one side, activation of PCD in an infected cell can curb virus replication, while some necrotic forms of PCD can assist spreading of virus to the surrounding tissue. In addition, certain PCDs can facilitate the identification of viruses by the adaptive immunity via emission of immunomodulatory secretomes that enhance antigen presentation [203]. To the infected host, activation of certain inflammatory PCDs may exacerbate disease pathogenesis, as these forms of PCD may enhance acute cytokine storms and/or potentially trigger delayed immunopathologies such as autoimmune disorders. Studies investigating how viral induced PCDs may instigate host reactivity against self are currently lacking.
